# Supplementary material for: Saccharomyces boulardii (CNCM I-745) improves intestinal damage in sepsis by remodeling intestinal flora structure
Source: Front Cell Infect Microbiol. 2025 Jul 31;15:1602792. doi: 10.3389/fcimb.2025.1602792 (PMC12350329; doi:10.3389/fcimb.2025.1602792)
Supplement: Supplementary file 1 [file DataSheet1.zip › summary/summary/8_Advanced_analyse/2_PICRUSt2_result/METACYC/Group.DW0_vs_BW0/PICRUSt2_METACYC_Group.DW0_vs_BW0_diff.pdf]

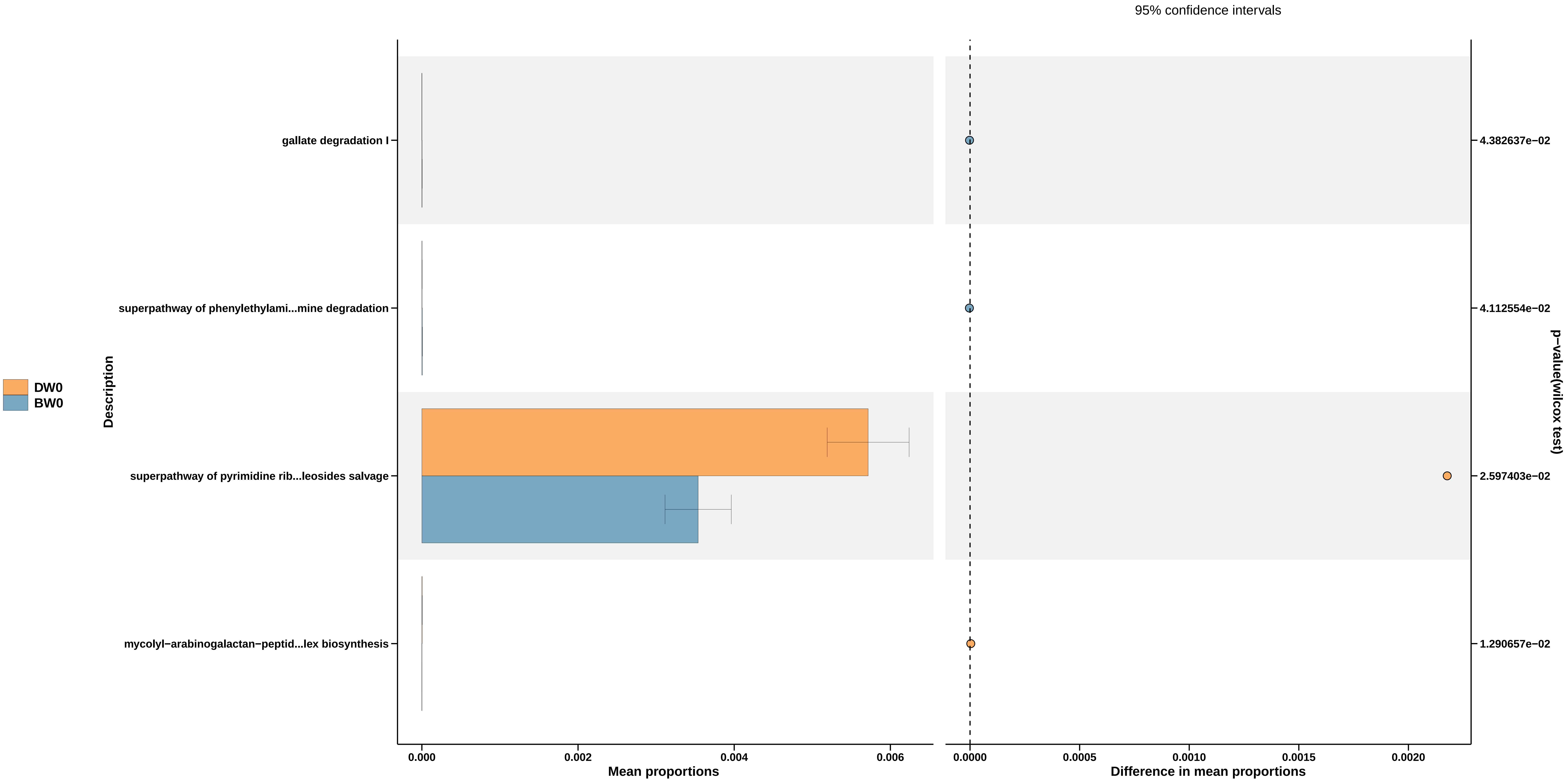

95% confidence intervals
gallate degradation I
superpathway of phenylethylami...mine degradation
superpathway of pyrimidine rib...leosides salvage
mycolyl−arabinogalactan−peptid...lex biosynthesis
Description
DW0
BW0
0.000
0.002
0.004
0.006
Mean proportions
0.0000
0.0005
0.0010
0.0015
0.0020
Difference in mean proportions
4.382637e−02
4.112554e−02
2.597403e−02
1.290657e−02
p−value(wilcox test)
